# The characteristics of thoracic aortic dissection in autopsy-diagnosed individuals: An autopsy study

**DOI:** 10.3389/fcvm.2022.973530

**Published:** 2022-10-11

**Authors:** Qianhao Zhao, Kun Yin, Nan Zhou, Qiuping Wu, Yuxi Xiao, Jinxiang Zheng, Da Zheng, Qiming Bi, Li Quan, Bingjie Hu, Jianding Cheng

**Affiliations:** ^1^Faculty of Forensic Medicine, Zhongshan School of Medicine, Sun Yat-sen University, Guangzhou, China; ^2^Guangdong Province Translational Forensic Medicine Engineering Technology Research Center, Sun Yat-Sen University, Guangzhou, China; ^3^Division of Forensic Medicine, Department of Pathology, School of Basic Medical Sciences, Guangzhou Medical University, Guangzhou, China; ^4^Department of Molecular Pathology, The Affiliated Cancer Hospital of Zhengzhou University, Zhengzhou, China; ^5^Henan Key Laboratory of Molecular Pathology, Zhengzhou, China

**Keywords:** thoracic aortic dissection, sudden death, forensic autopsy, epidemiology, retrospective study

## Abstract

Thoracic aortic dissection (TAD) is the most common cause of sudden cardiac death associated with aortic diseases. The age of TAD victims in forensic studies is significantly younger than hospitalized patients with TAD, while only a few studies have been conducted on autopsy-diagnosed TAD deceased. A retrospective study was conducted at the Medicolegal Center of Sun Yat-sen University from 1999 to 2019 to address the characteristics of TAD victims. A total of 200 deceased from spontaneous rupture of TAD were assessed, with 165 (82.5%) males and 175 (87.5%) Stanford type A deceased. Our main results showed that compared with patients with TAD diagnosed during their lifetime, individuals diagnosed with TAD until an autopsy showed an earlier onset (43.80 years old) and less accompanied hypertension (<50%). Sudden death was the initial symptom of 32 decedents. Instead of chest/back pain (40 decedents), abdominal pain (59 decedents) was the most common initial symptom, and 42 decedents presented with no accompanying pain. A higher proportion of abdominal pain and the painless symptom was associated with a higher risk of misdiagnosis. Women showed a more atypical clinical presentation and rapid progression than men. Younger decedents showed more pronounced left heart changes. The present study implicated the TAD individuals diagnosed until an autopsy as a particular entity, indicating the urgent need for further investigation on early diagnosis and pathogenesis of patients with TAD with atypical pain and painless or with younger age to reduce the burden of TAD-related sudden death.

## Introduction

Thoracic aortic dissection (TAD) is an emergent and life-threatening cardiovascular disease resulting in sudden cardiac death (SCD). Hospital studies have ascertained the annual incidence of acute aortic dissection (AAD) of about three cases per 100,000 (1), which may underestimate the incidence of aortic dissection as hospital-based reports do not account for preadmission deaths (2–5). Thus, these deaths are not accounted for unless an autopsy is performed. Systematic and standardized autopsy investigation is crucial for forensic pathologists to determine the cause of death of SCD victims (6). Our previous study reported that rupture of aortic dissection accounts for 5.2% of SCD victims (7). Prakash et al. (8) found that the age of TAD decedents was significantly younger than hospitalized patients with TAD, which indicated that autopsy-diagnosed TAD decedents might be a particular entity in TAD cases. However, only a few studies have been conducted on TAD deceased, and the sample size was too small to further investigate. Large sample size study of TAD deceased who were diagnosed until an autopsy could help us to uncover the nature of the particular TAD entity.

Our study recruiting 200 autopsy cases of TAD showed the characteristics of TAD deceased and identified that forensic autopsy is essential for determining the cause of death in sudden death cases and provides valuable additional information for physicians to further understand TAD. The prompt investigation of forensic autopsy data is indispensable to revealing pivotal information about TAD.

## Methods

### Study subjects

A retrospective study of forensic autopsy cases at the Medicolegal Center of Sun Yat-sen University was conducted. Autopsy reports that listed the primary cause of death as spontaneous rupture of TAD from 1999 to 2019 were reviewed. Decedents who died due to traumatic, iatrogenic TAD, or for who without a complete autopsy were excluded. Thus, 200 adult cases (≥18 years old) who met the study inclusion criteria formed the TAD group. As for control cases, each decedent from the TAD group was sex- and age-matched (same sex, with an age difference of no more than 3 years, with a death time difference of no more than 2 years) with three separate cases and decedents with aorta-related disease (intramural hematoma, penetrating aortic ulcer, aortic pseudoaneurysm, thoracic aortic aneurysms, abdominal aortic aneurysm/dissection, and aortic tumors) were excluded. All the control cases, aggregate up to 600, were classified into the control group. All the procedures followed were in accordance with the ethical standards of Sun Yat-sen University (approval no. 2019-004).

### Data collection

We retrospectively collected the index event's circumstances, including demographic data and medical history. Pathological data, including the location and extent of TAD, myocardial characteristics and dimensions, aortic and epicardial atherosclerotic diseases, and other congenital disabilities, was obtained from autopsy reports.

### Statistical analysis

Continuous variables were presented as mean ± SEM or median (interquartile range) and analyzed using the unpaired Student's *t*-test or Mann–Whitney *U*-test. Categorical variables were expressed as the count (percent) and analyzed using the Pearson's chi-squared test and Fisher's exact test. A measure probability value of *P* <.05 was considered significant. Statistical evaluation was performed using SPSS Statistics version 22.0 (IBM Incorporation, Chicago, Illinois, USA).

## Results

### Demographics

Overall, 200 deaths from spontaneous rupture of TAD were identified in a population aged from 19 to 73 years old, and the average age was 43.80 ± 0.75 years old; a total of 165 cases (82.5%) were male (Table 1). The TAD decedents in this study spanned more than 20 years, with significantly more decedents in the latter decade (149 decedents, 2010–2009) than in the first decade (51 decedents, 1999–2009). Still, there were no significant differences in the mean age at death, age distribution, and sex distribution between the two decades.

**Table 1:** Demographic characteristics of TAD cases.

	**Total (*n* = 200)**	**Male (*n* = 165)**	**Female (*n* = 35)**	***p*-value**
Age (Mean ± SD)	43.80 ± 0.75	43.75 ± 0.82	44.11 ± 10.67	0.8539
Age distribution [*n* (%)]				
19–30	22 (11)	18 (10.91)	4 (11.43)	0.941
31–40	55 (27.5)	47 (28.48)	8 (22.86)	
41–50	74 (37)	59 (35.76)	15 (42.86)	
51–60	37 (18.5)	31 (18.79)	6 (17.14)	
61–73	12 (6)	10 (6.06)	2 (5.71)	
Stanford type [*n* (%)]				
Stanford A	175 (87.5)	142 (86.06)	33 (94.29)	0.2622
Stanford B	25 (12.5)	23 (13.94)	2 (5.71)	

The TAD decedent number during the warmer period (85 decedents, May to October) in Southern China was less than that during the colder period (115 decedents, November to April) in our study. However, 60% (21 decedents) of female TAD died during the warm period, compared with only 38.32% (64 decedents, *P* = 0.0211) of the total male TAD deaths during the same period.

### Clinical data

Unfortunately, thirty-two victims (16%) have not been able to get to the hospital, and sudden death was their initial symptom (female: 8 in 35, 22.86%; male: 24 in 165, 14.55%). Meanwhile, the rest victims died very soon after admission to the emergency room. A total of 168 (84%) intervals from hospital admission to clinical death of TAD deaths were recorded. Seventy-one decedents (42.26%) died within 12 h, and 102 decedents (60.71%) died within 24 h. The interval from hospitalization to clinical death (IHD), with 17.5 (5.84, 41.21) h in men and 10.17 (3.33, 32) h in women, revealed a trend (*P* = 0.1966) in favor of men, and the median absolute difference of 7.33 h was likely to reveal the more urgent situation for women.

Excluding the TAD cases with sudden death as the initial symptom, abdominal pain was the most common symptom [59 decedents, 35.12% (Table 2)]. Rightfully, the most common misdiagnosis was acute abdomen. The acute onset of severe chest/back pain was the second most common initial complaint (40 decedents, 23.81%). In addition, the TAD deceased with headache or low back pain as their initial symptom showed a lower TAD suspected diagnosis ratio and a shorter IHD than those initially with chest/back pain or abdominal pain (Table 2). Meanwhile, 42 decedents (25%) presented with no accompanying pain in the present study. Syncope, chest distress, distention, and hemiplegia or paresis were the primary manifestations among these painless TAD deceased (Table 2). Painless TAD deceased showed a lower TAD suspected diagnosis ratio [4.76 vs. 12.70% (Table 2)] and a shorter IHD [10.98 (3.20, 30.00) vs. 18.67 (6, 42.09) (Table 2)] than the painful TAD deceased. Surprisingly, the proportion of painless victims was slightly higher among females than males (40.74 vs. 21.99%, *P* = 0.0519).

**Table 2:** Initial symptoms of TAD deceased whose prior symptom was not sudden death.

**Initial symptoms**	**Total (*n* = 168)** **n (%)**	**Suspected TAD (*n* = 18)** ***n* (%)**	**IHD^$^ (hours) Median (IQR)**	***p*-value**
Pain	126 (75)	16 (12.70)	18.67 (6, 42.09)	0.0716^*^
Headache	9 (5.36)	0	9 (3.20, 11.84)	0.0114^#^
Chest pain or back pain	40 (23.81)	9 (22.5)	24.93 (10.72, 69.93)	
Abdominal pain	59 (35.12)	7 (11.86)	19 (5.63, 39.98)	
Low back pain	18 (10.71)	0	9.71 (5.08, 26.00)	
Painless	42 (25)	2 (4.76)	10.98 (3.20, 30.00)	
Dizziness or syncope	13 (7.74)	1 (7.69)		
Chest distress	8 (4.76)	0		
Distention or emesis	7 (4.17)	0		
Hemiplegia or paresis	6 (3.57)	1 (16.67)		
Cold	8 (4.76)	0		

$ Interval from hospitalization to clinical death.

* p-value of intervals between painful deceased and painless deceased.

# p-value of intervals within painful deceased.

The clinical characteristics and outcomes in deceased with TAD vary with age. Compared with the older TAD deceased [>35 years old, 157 decedents, 47 (41, 52) years old], the incidence of sudden death as the initial symptom in youngers [≤35 years old, 43 decedents, 30 (26, 33) years old] was significantly higher (27.91 vs. 12.74%, *P* = 0.0319), while the incidence of painless TAD deceased was significantly lower among younger victims than older victims (6.45 vs. 29.20%, *P* = 0.0061).

Hypertension is an independent risk factor for TAD. In the present study, only 55.17% (32 in 58 available cases) of TAD deceased had a history of hypertension, and 48.74% (58 in 119 available cases) of TAD deceased with systolic blood pressure (SBP) ≥140 mm Hg.

### Autopsy findings

Autopsy findings are shown in Table 3. According to Stanford classification, Stanford type A (175 cases, 87.5%) deceased was much more common than Stanford type B (25 cases, 12.5%). Dissection extended to the abdominal aorta in 41 victims (type A: 35). The intimal tear was most often single. Only in 8 cases, there were two tears, and in 1 case, there were three tears. The intimal tear was mainly found at ascending aorta in type A decedents, while at descending aorta in type B decedents, which was the same with the adventitial tear. Therefore, cardiac tamponade due to a rupture of the dissected channel into the pericardial sac was the most common cause of death for type A TAD decedents (167 of 175, 95.43%).

**Table 3:** Postmortem findings in TAD deceased.

**Postmortem findings**	**Total *n* = 200** ***n* (%)**	**Type A *n* = 175** ***n* (%)**	**Type B *n* = 25** ***n* (%)**
Entry tear			
Ascending aorta	145 (72.5)	145 (82.86)	0 (0)
Arch of aorta	37 (18.5)	25 (14.29)	12 (48)
Descending aorta	24 (12)	10 (5.71)	14 (56)
Exit tear			
Ascending aorta	165 (82.5)	165 (94.29)	0 (0)
Arch of aorta	13 (6.5)	4 (2.29)	9 (36)
Descending aorta	24 (12)	7 (4)	17 (68)
Cardiac tamponade	167 (83.5)	167 (95.43)	0 (0)
Thoracic hemorrhage	56 (28)	32 (18.29)	24 (96)
MFS	1 (0.5)	1 (0.57)	0 (0)
BAV	8 (4)	8 (4.57)	0 (0)
Coronary atherosclerosis	45 (22.5)	42 (24)	3 (12)
Renal cyst	16 (8)	14 (8)	2 (8)
Heart weight (g)	450 (400, 500)	450 (400, 510)	400 (355, 485)

Pathological examination of the hearts from most TAD autopsy cases showed severe ventricular hypertrophy with an average cardiac mass of 450 (400, 500) g [control: 360 (300, 410) g, *P* < 0.0001 (Table 4)] and an average left ventricular-free wall thickness of 1.4 (1.2, 1.5) cm [control: 1.2 (1.1, 1.4) cm, *P* < 0.0001 (Table 4)]. As for the circumference of the cardiac valve annulus, except for the tricuspid annulus, the circumferences of the mitral/aortic/pulmonary annulus in the TAD group were significantly longer than those in the control group. Furthermore, we found that younger TAD decedents showed more pronounced changes in the left heart than their control counterparts. Compared with their control counterparts, younger TAD decedents not only had thicker left ventricular free wall [1.4 (1.2, 1.5) vs. 1.2 (1.0, 1.3) cm, *P* < 0.0001 (Table 5)] and longer circumference of the aortic annulus (CAA) [7.8 (7, 8.5) vs. 6.5 (6, 7) cm, *P* < 0.0001 (Table 5)], but also had a longer circumference of the mitral annulus (CMA) [9.7 (9, 10.5) vs. 9 (8.5, 9.5) cm, *P* = 0.0007 (Table 5)]. In addition, the differences in heart weight, CMA, and CAA between younger TAD and their control counterparts were more significant than those between older TAD and their control counterparts [heart weight: 90 vs. 70 g, CMA: 0.7 vs. 0 cm, and CAA: 1.3 vs. 0.5 cm (Table 5)].

**Table 4:** Postmortem finding differences between TAD deceased and control counterparts.

	**TAD (*n* = 200)**	**Control (*n* = 600)**	***p*-value**
Height (cm)	169 (163, 173)	165 (160, 170)	<0.0001
Heart weight (g)	450 (400, 500)	360 (300, 410)	<0.0001
TLV (cm)	1.4 (1.2, 1.5)	1.2 (1.1, 1.4)	<0.0001
TRV (cm)	0.3 (0.3, 0.4)	0.3 (0.3, 0.3)	<0.0001
CMA (cm)	9.5 (9, 10)	9.2 (8.5, 10)	0.0107
CTA (cm)	11.5 (11, 12)	11.5 (11, 12.23)	0.9304
CAA (cm)	7.5 (7, 8)	7 (6.5, 7.5)	<0.0001
CPA (cm)	8 (7.5, 8.5)	7.55 (7, 8)	0.0208
Coronary atherosclerosis (%)	45 (22.5)	99 (16.5)	0.0703
Renal cyst (%)	16 (8)	22 (3.67)	0.0197

TLV, Thickness of left ventricular wall; TRV, Thickness of right ventricular wall; CTA, Circumference of tricuspid annulus; CPA, Circumference of pulmonary annulus; CMA, Circumference of mitral annulus; CAA, Circumference of aortic annulus.

**Table 5:** Differences of postmortem findings between different age groups and their control counterparts.

**Age≤35**	**TAD** **(*n* = 43)**	**CTR** **(*n* = 129)**	***p*-value**	**Age>35**	**TAD** **(*n* = 157)**	**CTR** **(*n* = 471)**	***p*-value**
Height (cm)	172.57 ± 1.37	167.03 ± 0.63	0.0005	Height (cm)	168 (161, 172)	165 (160, 170)	0.003
Heart weight (g)	400 (360, 460)	310 (280, 380)	<0.0001	Heart weight (g)	450 (400, 515)	380 (300, 420)	<0.0001
TLV (cm)	1.4 (1.2, 1.5)	1.2 (1.0, 1.3)	<0.0001	TLV (cm)	1.4 (1.2, 1.55)	1.3 (1.1, 1.4)	<0.0001
TRV (cm)	0.3 (0.3, 0.4)	0.3 (0.3, 0.3)	0.0089	TRV (cm)	0.3 (0.3, 0.4)	0.3 (0.3, 0.4)	0.0003
CMA (cm)	9.7 (9, 10.5)	9 (8.5, 9.5)	0.0007	CMA (cm)	9.5 (9, 10)	9.5 (9, 10)	0.332
CTA (cm)	11.5 (11, 13)	11.5 (11, 12)	0.2391	CTA (cm)	11.5 (11, 12)	11.8 (11, 12.5)	0.4046
CAA (cm)	7.8 (7, 8.5)	6.5 (6, 7)	<0.0001	CAA (cm)	7.5 (7, 8)	7 (6.5, 7.5)	<0.0001
CPA (cm)	8 (7, 8.5)	7.5 (7, 8)	0.0151	CPA (cm)	8 (7.5, 8.5)	8 (7.2, 8.5)	0.2147

**Figure 1:**
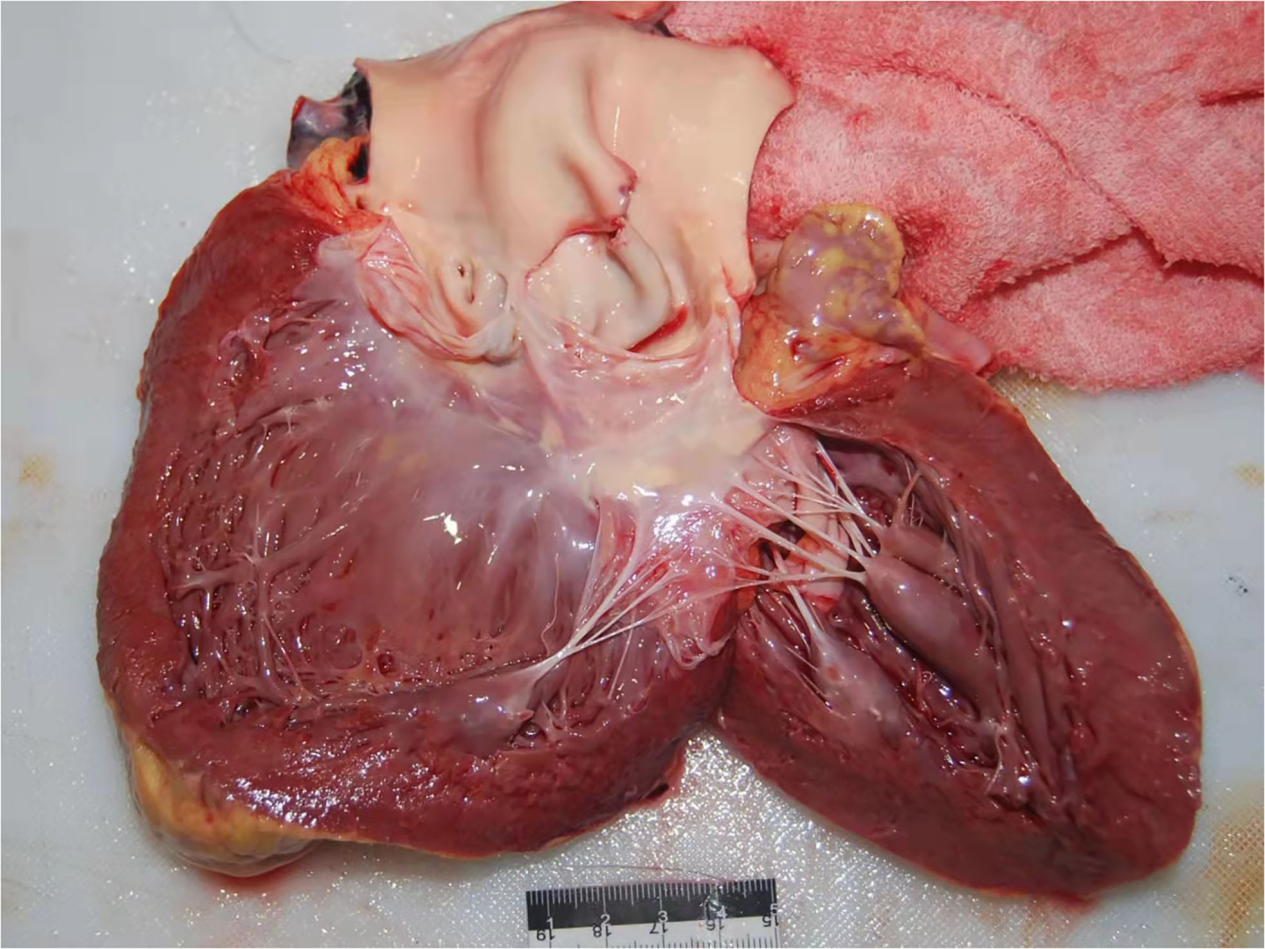
Representative picture of bicuspid aortic valves.

**Figure 2:**
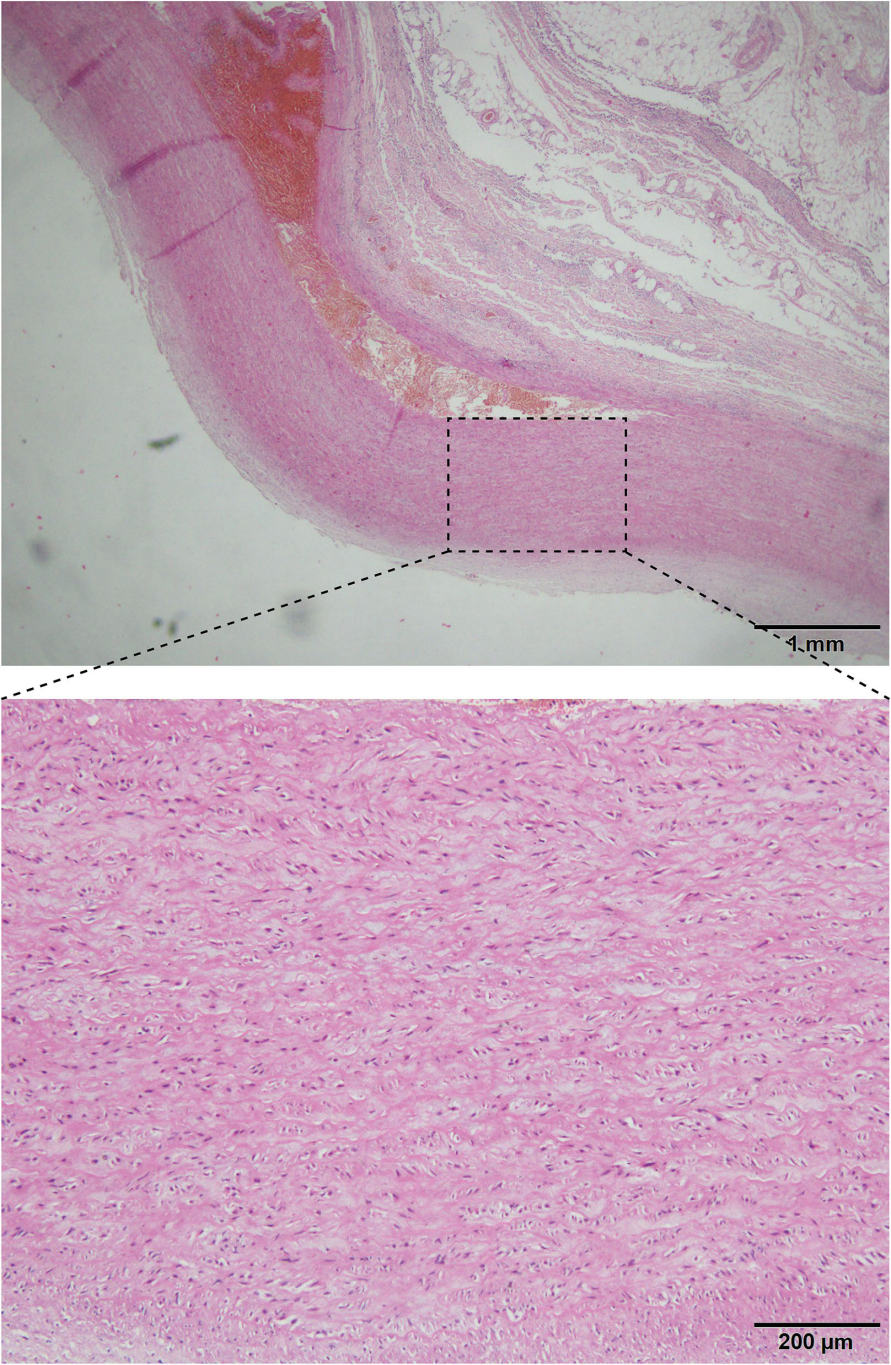
Aorta of a TAD decedent.

The most common congenital disorder found in this study was bicuspid aortic valves (BAV) [eight cases, 4%, all Stanford A (Table 3, Figure 1)], classified by autopsy. Only one deceased was found with Marfan syndrome (MFS). The prevalence of coronary atherosclerosis in the TAD group was slightly higher than in controls (22.5 vs. 16.5%, *P* = 0.0703), but generally, there is no association of coronary atherosclerosis with TAD. Patients with TAD were reported to have a greater prevalence of renal cysts on CT than healthy controls. Our findings regarding the detection rate of renal cysts in TAD deceased were consistent with this [8 vs. 3.67%, *P* = 0.0197 (Table 3)]. As to microscopic examination of medial alteration, the mucoid extracellular matrix accumulation (MEMA) was confirmed in all the available cases to varying grades (Figure 2).

In addition to clinical data, a forensic pathological autopsy could also find some anatomical features that might be related to hypertension, such as the thickness of the compact myocardium of the left ventricular >1.5 cm, heart weight >500 g, hyalinosis of the central splenic artery, and renal arteriosclerosis. However, none of the above indicators had a positive rate of more than 50% alone (Table 6).

**Table 6:** Hypertensive indicators in TAD deceased.

**Indicator of hypertension**	***n* (%)**
History of hypertension^*^	32 (55.17)
SBP ≥140 mmHg^#^	58 (48.74)
Thickness of left ventricular wall ≥1.5 cm	48 (24)
Heart weight >500 g	47 (23.5)
Hyalinosis of central splenic artery	86 (43)
Renal arteriolosclerosis	54 (27)
All negative	39 (19.5)

* Only 58 cases with available record of hypertension.

# Only 119 cases with available records of blood pressure.

## Discussion

Thoracic aortic dissection still represented one of the most catastrophic cardiovascular diseases. Hitherto, many series of clinical studies have been conducted on hospitalized patients with TAD. Only reporting the incidence of hospitalized patients would introduce a serious underestimation of the incidence. A large number of patients with TAD remained undiagnosed until postmortem forensic autopsy and were not included in routine clinical studies of TAD (9–11). TAD decedents diagnosed until autopsy generally experienced fewer medical interventions and had a more natural course and a more severe outcome than hospitalized patients with TAD. However, studies on the deceased with TAD are limited. Our study recruited 200 autopsy-diagnosed TAD decedents and found that the characteristics of TAD among individuals diagnosed until autopsy were significantly different among hospitalized patients with TAD.

### Early onset and low prevalence of hypertension

It has been shown in several studies that Chinese patients with AD are approximately 10 years younger than patients from western countries (12, 13). Similarly, compared to the TAD victims in the west, the age of TAD victims in our study was significantly younger (8, 14, 15), which may be related to the inadequate control of hypertension in China. Furthermore, compared with the age of onset of clinical patients with TAD, the age of TAD victims in forensic TAD studies was significantly younger (12, 13, 15, 16). Similar findings have been found in studies abroad between forensic and clinical TAD cases (8, 17). One possible reason was that the older patients were more likely to seek medical care after the onset of symptoms and be admitted with higher blood pressure. However, advanced age was associated with less administration of forensic autopsy. Earlier onset of TAD deceased in our study brings up a lower incidence of comorbid hypertension. In addition to those well-known possible causes such as incomplete clinical data, poor health awareness, and fewer routine physical examinations, the high proportion of Stanford type A cases may be another reason for the low prevalence of hypertension in our study, for the reason that hypertension was more prevalent in those with type B AD than in those with type A AD (13, 18, 19).

### Atypical pain and painless makes thoracic aortic dissection much more atypical

Aortic dissection has a myriad of clinical presentations and is for certain a diagnostic challenge. The symptoms of patients with TAD can vary and sometimes be very minimal. Symptoms of chest pain, abdominal pain, low back pain, limb ischemia, neurological dysfunction, and syncope have all been reported as potentially the sole presentation of TAD (20–23). In common sense, the most common symptom of TAD is chest pain, which is usually sharp and sometimes reported as tearing or ripping, while often radiating to the back or the abdomen (18, 24). Acute aortic chest pain can mimic pain from other more common acute conditions such as angina and acute coronary syndrome (ACS), which is more common than TAD and is, therefore, preferentially suspected by clinicians (20). The proportion of patients eventually diagnosed with TAD who receive a prior emergency diagnosis and management plan for ACS can be as high as 80% (25).

While the most common symptom in the present study was the sudden onset of abdominal pain rather than chest pain, regardless of Stanford type, which was different from that reported by the International Registry of Acute Aortic Dissection (IRAD) (chest pain) (24) and Sino-RAD (back pain) (13). Owe to lesion location, it is easy to understand that most Stanford type B patients with TAD presented with pain in the abdomen or low back. However, abdominal pain was also the initial symptom in Stanford type A deceased in the present study. Upchurch et al. found that patients who presented primarily with acute abdominal pain alone (4.6%) tended to have a delayed diagnosis and had higher mortality than those with more typical symptoms (26). In addition, our study found that TAD deceased present primarily with headache or low back pain showed a lower rate of suspected TAD diagnosis and a shorter IHD than those present with chest/back pain (Table 2). Atypical pain symptoms make correct diagnosis difficult.

Sudden onset of severe chest, back, or abdominal pain represents the most frequent symptom (13, 24). However, atypical presentation is seen in ~5% of AAD and is characterized by painless aortic dissection associated with higher mortality (27–29). In our study, the figure was 25%, far more than that in other studies. The trend of lower TAD suspected diagnosis ratio and shorter IHD in painless TAD decedents implicated that TAD decedents with painless symptoms propagated more rapidly and were more vitally. The higher risk of misdiagnosis secondary to atypical manifestations might be a crucial cause of the death of patients with TAD.

### Female: More atypical and critical

Women were reported to be about 5 years older than men at presentation in hospitalized patients with TAD (10, 30–32). In the present autopsy-based study, women were of comparable age to men and did not show longer event-free survival (Table 1). Interestingly, we found that women were more frequently affected during the warmer period, which was not the higher risk season for TAD (33, 34). Besides, women also showed a higher proportion of painless symptoms. These atypical features of TAD in women could mislead clinicians about the diagnosis. Further, our study found that women showed a higher proportion of sudden death as an initial symptom and shorter IHD. These results indicated that women die faster once the onset. Atypical clinical presentation and rapid progression made women with dissection die more often than men. Similar findings have been suggested in other hospitalized patient-based studies (10, 31) and speculated that sex differences in aortic morphology and hidden coronary heart disease (CHD) in women were possible reasons (35). In our study, female and male TAD decedents showed a comparable proportion of CHD. Compared to their control counterparts, women were more frequently affected by CHD (women, TAD vs. control: 22.86 vs. 5.71%; men, TAD vs. control: 22.42 vs. 18.79%), which may provide a clue for the early diagnosis of TAD in women.

### Younger thoracic aortic dissection decedents show more pronounced left ventricular hypertrophy

Left ventricular hypertrophy (LVH) was previously identified in AAD using echocardiography (36–38), and LVH was also present in the youngest subjects with AAD and did not increase in relationship to age (8). Interestingly, in our study, younger TAD decedents with a higher prevalence of normotension showed more pronounced morphological changes in the left heart (Table 5). This result suggested that monitoring of cardiac volumes by echocardiography may be helpful in the early screening of TAD, especially in young patients. Further, familial TAD caused by several genetic mutations was also reported to be associated with disproportionate LVH in normotensive individuals (39), raising the possibility that some LVH (especially in youngers) may result from a genetic predisposition for both TAD and cardiac disease. In addition, our previous study found that the more genetic variants an individual carries, the earlier the onset of dissection (16). Genetic screening would help to anticipate prognosis and establish accurate risk stratification of any family member at risk of developing the same disease, especially for those of young TAD deceased.

### Limitation

Due to the retrospective nature of the present autopsy study, the body mass index (BMI) data in some autopsies was inadequate, which is associated with mortality in patients with cardiovascular diseases (CVDs) (40, 41) and is very important to evaluate the risks of CVD. Besides, as we do not routinely conduct toxicological analysis in decedents with a clear cause of death, cocaine use is not available in this study, which is associated with numerous CVDs and SCD (42, 43). Furthermore, there was no adequate correlation between postmortem values and values obtained from living patients by imaging techniques. Last, our results relied on data from a single center that may not reflect nationwide trends.

## Conclusion

In summary, a significant number of patients remained undiagnosed until postmortem autopsy and were rarely reported compared to hospitalized patients. We conducted an autopsy-based study of TAD to detect the characteristics of the TAD deceased. Our results implicated the TAD individuals diagnosed until an autopsy was a particular entity. It showed an earlier onset and a low incidence of hypertension, a high incidence of painless and abdominal pain, more atypical and critical situation for women, and more pronounced left heart changes for young decedents. Our findings indicate the urgent need for studies on forensic TAD deaths to explore the early diagnosis of atypical TAD, as well as the pathogenesis and prevention of young patients with TAD to minimize the burden of TAD-related sudden death.

## Data availability statement

The original contributions presented in the study are included in the article, further inquiries can be directed to the corresponding author/s.

## Ethics statement

This project was approved by the Ethics Committee of Sun Yat-sen University (Approval No. 2019-004), and it was carried out in strict accordance with the ethical research principle of Sun Yat-sen University.

## Author contributions

JC, BH, and QZ designed the project. QZ, NZ, QW, YX, and JZ collected all the data. DZ and QB managed the documents and tissue slides. KY managed the histopathological data. QZ, NZ, and QW analyzed the data. QZ, LQ, BH, and JC discussed the data. QZ, BH, and JC wrote the manuscript. All the authors have approved the final version of the manuscript.

## Funding

This study was supported by the National Natural Science Foundation of China (grant nos. 81920108021 and 81901919), the Guangzhou Municipal Science and Technology Project (grant no. 202102080092), and the Natural Science Foundation of Guangdong Province (grant no. 2020A1515010078).

## Conflict of interest

The authors declare that the research was conducted in the absence of any commercial or financial relationships that could be construed as a potential conflict of interest.

## Publisher's note

All claims expressed in this article are solely those of the authors and do not necessarily represent those of their affiliated organizations, or those of the publisher, the editors and the reviewers. Any product that may be evaluated in this article, or claim that may be made by its manufacturer, is not guaranteed or endorsed by the publisher.
